# Correlation between Clinical Characteristics and Chest Computed Tomography Findings of Pulmonary Cryptococcosis

**DOI:** 10.1155/2015/703407

**Published:** 2015-02-12

**Authors:** Hideaki Yamakawa, Masahiro Yoshida, Masami Yabe, Emiri Baba, Keitaro Okuda, Shota Fujimoto, Hiroaki Katagi, Takeo Ishikawa, Masamichi Takagi, Kazuyoshi Kuwano

**Affiliations:** ^1^Department of Internal Medicine, Division of Respiratory Medicine, Jikei University School of Medicine, Kashiwa Hospital, 163-1 Kashiwashita, Kashiwa, Chiba 277-8567, Japan; ^2^Division of Diagnostic Pathology, Jikei University School of Medicine, Kashiwa Hospital, Chiba, Japan; ^3^Department of Internal Medicine, Division of Respiratory Medicine, Jikei University School of Medicine, Tokyo, Japan

## Abstract

*Objective*. The aim of this study was to review HIV-negative patients with pulmonary cryptococcosis to analyze the correlations between clinical characteristics and chest computed tomography (CT) findings. *Methods*. We retrospectively analyzed medical records of 16 HIV-negative patients with pulmonary cryptococcosis diagnosed at our institution, and clinical characteristics of the patients with nodules or masses without ground-glass attenuation (GGA)/consolidation type were compared with those of patients with inclusive GGA or consolidation type. *Results*. Host status was immunocompromised (81.2%) in most of the patients, and 6 (37.5%) were asymptomatic. The most frequent radiologic abnormalities on chest CT scans were one or more nodules (87.5%), GGA (37.5%), and consolidations (18.8%). Most lesions were located in the lower lung. Levels of hemoglobin and platelets were significantly lower in patients with inclusive GGA or consolidation type. Although the differences were not significant, patients with inclusive GGA or consolidation type tended to have a C-reactive protein level of ≥1.0 mg/dL. *Conclusion*. If a patient with anemia and thrombocytopenia shows GGA or consolidation in the lung, pulmonary cryptococcosis should be given careful consideration.

## 1. Introduction

Cryptococcosis, which occurs sporadically worldwide, is a potentially serious fungal disease that is typically caused by inhalation of* Cryptococcus neoformans* or* C. gattii*, which tends to form an aerosol [1]. The fungus most commonly infects patients with human immunodeficiency virus (HIV) and other causes of reduced immunity, and it less frequently infects immunocompetent patients. The respiratory tract is thought to be the entry site and is the organ most frequently involved when cryptococcal infection develops [2, 3]. Radiographic presentations of pulmonary cryptococcosis are varied. Well-defined single or multiple pulmonary nodules are the most frequent radiologic abnormality, followed by pulmonary infiltrates. Furthermore, cavitation within nodules and parenchymal consolidation are more common in immunocompromised than in immunocompetent patients [3].

When chest radiographic abnormalities of a patient show consolidation and ground-glass attenuation (GGA), differential diagnosis with bacterial pneumonia and organizing pneumonia may be difficult, resulting in delay, and therefore clinicians must be careful to recognize that GGA or consolidation may be a radiological finding of pulmonary cryptococcosis [4]. Thus, in which patients should careful attention to pulmonary cryptococcosis be paid? Assessment of clinical and radiological features in pulmonary cryptococcosis patients may be useful because non-HIV patients with pulmonary cryptococcosis have a good prognosis with appropriate and early management. Lower levels of CD4+ lymphocytes in patients with HIV infection are a risk factor for cryptococcosis [5]. However, few investigations have compared the clinical characteristics and chest CT imaging findings of pulmonary cryptococcosis in non-HIV patients [6–8]. The aims of our study were thus to retrospectively review pulmonary cryptococcosis patients and assess the correlation between clinical characteristics and chest CT findings.

## 2. Material and Methods

### 2.1. Patients

We studied 16 HIV-negative patients with pulmonary cryptococcosis over 18 years of age who were diagnosed by histological or cytological presence of the organism in lung biopsy specimens, by positive findings from culture of respiratory specimens, or by a positive result of a serum cryptococcal antigen test with radiographic evidence of pulmonary disease. The patients were diagnosed from April 2006 through June 2014 at Jikei University School of Medicine, Kashiwa Hospital, Chiba, Japan.

### 2.2. Study Design

This was a retrospective study for which clinical data were collected from patient medical records. Baseline clinical measurements and chest CT findings were obtained within 7 days of the diagnosis of pulmonary cryptococcosis. A consensus reading of the CT images was conducted by two observers. All cryptococcal antigen titers were measured by means of commercially available latex agglutination test (Eiken, Tokyo, Japan). A concentration of greater than 6.25 ng/mL was considered the cut-off point for a positive result. This study was approved by the Institutional Review Board of Jikei University School of Medicine (number 26-200 (7705)).

### 2.3. Data Analysis

Data are presented as median [range] unless otherwise stated. Fisher's exact test or Mann-Whitney *U* test was used for between-group comparisons with nominal and continuous variables, respectively. A *P* value < 0.05 was considered statistically significant. Missing data were categorized as “unknown” and were entered into each statistical analysis model. All data were analyzed with SPSS version 22.0 for windows (IBM Japan, Tokyo, Japan).

## 3. Results

### 3.1. Patient Characteristics

Clinical characteristics of the 16 patients with pulmonary cryptococcosis are summarized in Table 1. Ten patients (62.5%) were men and 6 (37.5%) were women, with a median age of 60 [range: 25–82] years. Host status was immunocompromised in 81.2% of the patients, of whom 5 patients had hematologic malignancy, 3 patients received corticosteroid and/or other immunosuppressive drugs for myasthenia gravis, 2 patients had advanced solid tumor, 2 patients received corticosteroid for chronic nephritis, and 1 patient received renal transplantation. However, 87.5% of patients did not have impaired glucose tolerance (hemoglobin A1c (HbA1c) < 6.5%) and 6 (37.5%) were asymptomatic. Fever and cough were the most common presenting symptoms, followed by general fatigue. Elevation of the peripheral white blood cell (WBC) count was detected in 6 patients (37.5%) (normal range: 3200–9000/*μ*L). A lymphocyte cell count of <1000/*μ*L was present in 7 (43.8%) patients (4 with hematologic malignancy, 2 receiving corticosteroid, and 1 with solid tumor), a hemoglobin (Hb) of <10 g/dL in 7 (43.8%) patients (5 with hematologic malignancy, 1 with solid tumor, and 1 receiving corticosteroid), a platelet count (Plt) of <10.0 × 10^4^/*μ*L in 5 (31.3%) patients (all with hematologic malignancy), an albumin level of <3.0 g/dL in 6 (37.5%) patients (3 with hematologic malignancy, 2 with solid tumor, and 1 receiving corticosteroid), and a low immunoglobulin G (IgG) of <800 mg/dL in 6 (37.5%) patients (4 receiving corticosteroid and 2 with hematologic malignancy). All patients were positive for serum cryptococcal antigen titer. Cerebrospinal fluid (CSF) obtained via lumbar puncture was examined in 6 (37.5%) patients, and all cultures were negative. Only 1 patient was found to be positive for Grocott stain and cryptococcal antigen titer in CSF, which indicated meningitis.

The diagnosis of pulmonary cryptococcosis was made by direct tissue examination in 5 patients (transbronchial lung biopsy in 4 and video-assisted thoracoscopic surgery in 1) and by cytology from bronchial brushings and washing in 2 patients. One patient was positive for bronchial washing fluid culture, which showed* C. neoformans*. Cultures in the other patients did not prove the diagnosis. The other 9 (56.3%) patients were diagnosed clinically by the positive result of a serum cryptococcal antigen test with radiographic evidence of pulmonary disease. Treatment included antifungal drugs alone in 10 (62.5%) patients, surgery plus antifungal therapy in 1 (6.3%), and none in 4 (25.0%) patients. Of the 11 patients who received antifungal therapy, 10 patients improved and 1 died due to underlying disease during the follow-up period. Underlying disease in the 4 patients who received no treatment (hematological malignancy in 3 and pancreatic cancer in 1) progressed rapidly, and antifungal treatment was impossible.

### 3.2. Radiologic Features

**Figure 1:**
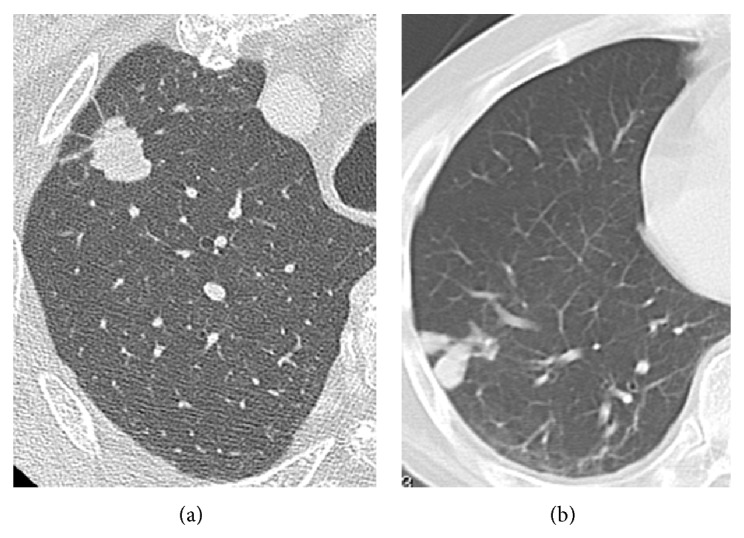
Representative chest CT images of pulmonary cryptococcosis of the nodule or mass without GGA/consolidation type. One patient showed a solid nodule with spiculation in the right upper lung (a). Another patient showed plural nodules, which had contact with the pleura in the right lower lobe (b).

**Figure 2:**
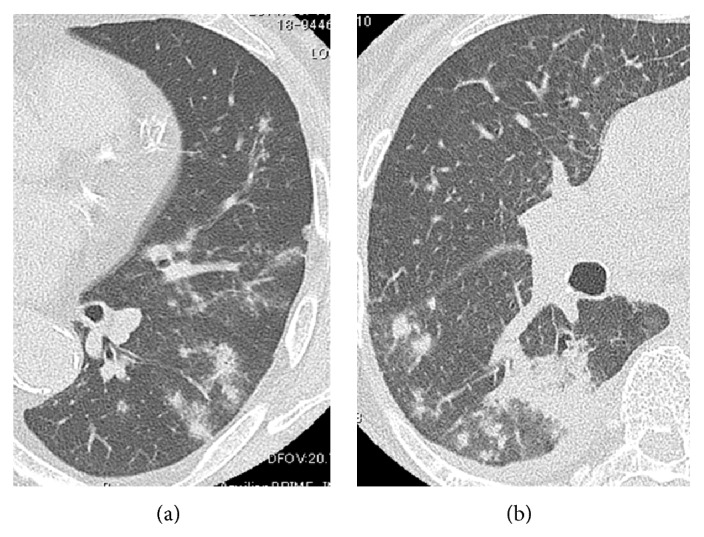
Representative chest CT images of pulmonary cryptococcosis in inclusive GGA or consolidation type. One patient showed multiple pale nodules with GGA in the left lung (a). Another patient showed multiple pale nodules, consolidation, and pleural effusion in the right lower lobe (b).

The pulmonary abnormalities seen on the initial CT scans at diagnosis are summarized in Table 2. The most common radiologic finding was the presence of one or more pulmonary nodules (87.5%). A solitary nodule was seen in 18.8% (Figure 1(a)) and multiple nodules were seen in 68.7% (Figure 1(b)) of patients. The second most common finding was GGA (37.5%) (Figure 2(a)). Other associated findings included consolidations (18.8%) (Figure 2(b)), pleural effusion (25.0%), and calcification (6.3%). Lesions in the right lung were present in 13 (81.3%) patients, in the left lung in 8 (50.0%) patients, and bilaterally in 5 (31.3%) patients, in whom the lesions were more frequent in the right lung. Ten (62.5%) patients had lesion involvement in the lower lobe, 6 (37.5%) patients had upper lobe involvement, and 3 (18.8%) patients had middle lobe involvement, which was more frequent in the lower lobe. Lesion extent was evaluated in the pulmonary lobes, including the upper, middle, and lower lobes in the right lung and the upper lingular and lower lobes in the left lung. Most lesions (62.5%) were located in one lobe, and no lesions were located in 4 or more lobes.

### 3.3. Correlation between Clinical Characteristics and Chest CT Findings

The clinical characteristics of the 7 patients with a nodule or mass without GGA/consolidation type were compared with those of the 9 patients with inclusive GGA or consolidation type and are summarized in Table 3. Neither sex, age, host status, body mass index (BMI), and symptoms at initial consultation nor HbA1c, WBC, lymphocytes, and IgG were significantly different between the two groups. However, Hb and Plt were significantly lower in patients with inclusive GGA or consolidation type (*P* = 0.044 and *P* = 0.023, resp.). Although the differences were not significant, patients with inclusive GGA or consolidation type tended to have a C-reactive protein level of ≥1.0 mg/dL (*P* = 0.060).

## 4. Discussion

Pulmonary cryptococcosis refers to acute or chronic infection of the lungs caused by inhalation of* Cryptococcus* species, which tend to form an aerosol. The diagnosis of pulmonary cryptococcosis is based on serological, etiological, and histological results. Lung tissue biopsies and pathological examinations are the main methods used to confirm the diagnosis. Most of our patients had a clinical diagnosis that was based on the positive result of a serum cryptococcal antigen test with radiographic evidence of pulmonary disease then had a definitive diagnosis based on direct tissue examination of cytology results. The serum cryptococcal antigen test is a highly specific test that is often used to diagnose cryptococcal infection [9], and, many times, invasive inspection of clinical sites of infection is impossible. Therefore, we thought it valuable to include patients diagnosed as having pulmonary cryptococcosis by all diagnostic methods.

Cryptococcosis typically occurs in immunocompromised patients such as those with HIV/AIDS, and the presence of pulmonary* Cryptococcus* sp. in HIV/AIDS patients is associated with high mortality [10]. The clinical descriptions of pulmonary cryptococcosis in HIV-negative individuals are quite limited because of the rarity of the disease itself. In general, males are more frequently infected than females, and, in the present study, the disease also occurred predominantly in males [11, 12]. Nadrous et al. reported that about one-third of immunocompetent patients with pulmonary cryptococcosis were asymptomatic [13]. Although the present study comprised predominantly immunocompromised patients (81.2%), a similar ratio of asymptomatic patients was also observed, indicating that even asymptomatic patients with immunodeficiency require attention.

The radiological findings of pulmonary cryptococcosis on chest CT are variable. Most of our patients (87.5%) had pulmonary nodules (solitary: 18.8%, multiple: 68.7%). The high frequency of these lesions is consistent with that reported by other studies [8, 11, 12, 14, 15]. The lesions were located predominantly in the lower lung (62.5%) rather than in the middle (18.8%) or upper lung (37.5%), which was also noted in other studies [11, 16–18], and were found mainly in the right (81.3%) rather than the left (50.0%) lung in most patients. Most lesions were found in a single lobe (62.5%), and no lesions were present in 4 or more lobes.

We investigated the correlation between clinical characteristics and chest CT findings in the patients with pulmonary cryptococcosis. GGA lesions and consolidations of pulmonary cryptococcosis appear to occur more frequently in immunocompromised hosts [3].* C. neoformans* is a facultative intracellular pathogen, and one important protective function against infection is cellular immunity [19]. Host defense against* C. neoformans* infection is mediated by Th-1-type cellular immunity, which is triggered by host cell recognition of the pathogen-associated molecular patterns via the pattern recognition receptors [20]. We found GGA lesions in 37.5% and consolidations in 18.8% of the patients. Inclusive GGA or consolidation type was more frequent in the immunocompromised hosts (88.9%), but the frequency of lesions without GGA/consolidation type in these hosts was nearly the same (71.4%). Generally, an “immunocompromised host” refers to a patient with malignant disease or one who has received an immunosuppression drug, has undergone an enforced transplantation, has severe diabetes, or is immunodeficient, but the definition of “immunocompromised host” is not yet clear. Therefore, we thought that the assessment of clinical data and radiological features related to pulmonary cryptococcosis may be useful because HIV-negative patients also have pulmonary cryptococcosis. Results of routine laboratory investigations in another study were generally nonspecific [7]. In our study, the levels of Hb and Plt were significantly lower in the patients with inclusive GGA or consolidation type than in those with a nodule or mass without GGA/consolidation type. Moreover, because there were no significant differences between the immunocompromised and nonimmunocompromised hosts, lymphocyte cell counts and IgG levels as simple indices of cellular and humoral immunodeficiency likewise were not significantly different between the two radiologic types. Therefore, the radiological shadows of pulmonary cryptococcosis in patients with anemia and thrombocytopenia might indicate GGA or consolidation. On the other hand, 5 patients in our study had hematologic malignancy, all of whom had anemia and thrombocytopenia. Further analysis should be done during anemia or thrombocytopenia follow-up of these patients with hematologic malignancy whose radiologic abnormality of pulmonary cryptococcosis tended to be of the inclusive GGA or consolidation type.

A limitation of this study is that it is a retrospective, single-site study with a small number of patients collected over a long period, and our results should not be generalized to the worldwide population. Despite this limitation, we conclude that if a patient with anemia and thrombocytopenia shows GGA or consolidation on radiological examination of the lung, pulmonary cryptococcosis should be given careful consideration. Additional studies are needed to further clarify the relation of clinical characteristics and radiological findings in pulmonary cryptococcosis.

## Figures and Tables

**Table 1:** Clinical characteristics of 16 patients with pulmonary cryptococcosis.

Characteristics	
Number of patients	16 (100.0%)
Male/female	10 (62.5%)/6 (37.5%)
Age (years)	60 [25–82]
<70 years/≥70 years	8 (50.0%)/8 (50.0%)
Host status	
Immunocompetent	3 (18.8%)
Immunocompromised	13 (81.2%)
BMI, kg/m^2^	20.8 [16.0–28.3]
<18.5, 18.5~25, ≥25 kg/m^2^	1 (6.3%)/12 (75.0%)/3 (18.7%)
HbA1c (%)	5.6 [4.9–7.0]
≥6.5%/<6.5%	2 (12.5%)/14 (87.5%)
Symptoms	
None	6 (37.5%)
Fever	5 (31.3%)
Cough	5 (31.3%)
General fatigue	3 (18.8%)
Dissemination/confined to the lung	1 (6.3%)/15 (93.7%)
Treatment and follow-up	
No treatment	4 (25.0%)
Antifungal drugs	10 (62.5%)
Surgery + antifungal drugs	1 (6.3%)
Unknown	1 (6.3%)

Laboratory data	Median [range]

WBC (/*μ*L)	8100 [1500–31400]
Lym (/*μ*L)	1300 [230–2900]
Hb (g/dL)	11.3 [4.4–16.5]
Plt (×10^4^/*μ*L)	18.0 [1.4–36.0]
Albumin (g/dL)	3.8 [3.2–4.1]
ESR (mm/hr)	27 [1–78]
CRP (mg/dL)	0.7 [0.1–7.0]
IgG (mg/dL)	889 [410–4121]
Positive for serum cryptococcal antigen titer	16 (100%)
Diagnosis by	
TBLB (histology)	4 (25.0%)
Surgery (histology)	1 (6.3%)
Bronchial brushing and washing (cytology)	2 (12.5%)
Positive of the serum cryptococcal antigen titer and radiological abnormalities	9 (56.3%)

Values are shown as median [range] or number (%).

BMI: body mass index, HbA1c: hemoglobin A1c, WBC: white blood cell, Lym: lymphocyte cell, Hb: hemoglobin, Plt: platelet, ESR: erythrocyte sedimentation rate, CRP: C-reactive protein, IgG: immunoglobulin G, and TBLB: transbronchial lung biopsy.

**Table 2:** Radiological patterns of pulmonary abnormalities of CT scans.

Abnormality	
Pulmonary nodules	14 (87.5%)
Solitary	3 (18.8%)
Multiple	11 (68.7%)
5–10	5 (31.3%)
>10	0 (0%)
Ground-glass attenuation	6 (37.5%)
Consolidations	3 (18.8%)
Pleural effusion	4 (25.0%)
Calcification	1 (6.3%)
Lesion area	
Right lung	13 (81.3%)
Left lung	8 (50.0%)
Bilateral lungs	5 (31.3%)
Lesion area	
Upper lung	6 (37.5%)
Middle lung	3 (18.8%)
Lower lung	10 (62.5%)
Extent of lesions (6 pulmonary lobes)	
1/2/3	10 (62.5%)/5 (31.3%)/1 (6.3%)
>4	0 (0%)

**Table 3:** Comparison of clinical characteristics between nodule or mass without GGA/consolidation type and inclusive GGA or consolidation type.

Clinical characteristics	Nodule or mass without GGA/consolidation type	Inclusive GGA or consolidation type	*P* value
Number of patients	7 (100.0%)	9 (100.0%)	
Male	5 (71.4%)	5 (55.5%)	0.633
Age (years), median [range]	73 [25–82]	60 [56–79]	0.873
≥70 years	4 (57.1%)	4 (44.4%)	>0.999
Host status			
Immunocompromised	5 (71.4%)	8 (88.9%)	0.550
BMI, kg/m^2^, median [range]	19.6 [16.0–24.0]	22.1 [17.6–25.0]	0.153
<18.5 kg/m^2^	2 (28.6%)	1 (11.1%)	0.550
Symptoms			
None	3 (42.9%)	3 (33.3%)	>0.999
HbA1c (%), median [range]	5.8 [5.1–6.5]	5.5 [4.9–7.0]	0.943
WBC (/*μ*L), median [range]	7400 [6400–12300]	8100 [1500–31400]	0.427
Lym (/*μ*L), median [range]	1300 [600–2900]	600 [230–2200]	0.265
<1000/*μ*L	2 (28.6%)	5 (55.6%)	0.358
Hb (g/dL), median [range]	12.0 [8.9–16.5]	8.4 [4.4–14.9]	**0.044**
<10.0 mg/dL	1 (14.3%)	6 (66.7%)	0.060
Plt (×10^4^/*μ*L), median [range]	24.6 [10.2–36.0]	8.4 [1.4–30.3]	**0.023**
<10.0 × 10^4^/*μ*L	0 (0%)	5 (55.6%)	**0.034**
Albumin (g/dL), median [range]	3.3 [2.5–4.9]	2.9 [2.4–3.9]	0.184
<3.0 g/dL	1 (14.3%)	5 (55.6%)	0.145
ESR (mm/hr), median [range]	10 [1–62]	74 [10–78]	0.176
CRP (mg/dL), median [range]	0.5 [0.1–7.0]	1.4 [0.1–6.7]	0.310
≥1.0 mg/dL	1 (14.3%)	6 (66.7%)	0.060
IgG (mg/dL), median [range]	885 [467–2507]	983 [410–4121]	0.897
<800 mg/dL	3 (42.9%)	3 (37.5%)	>0.999

GGA: ground-glass attenuation; BMI: body mass index; HbA1c: hemoglobin A1c; WBC: white blood cell; Lym: lymphocyte cell; Hb: hemoglobin; Plt: platelet; ESR: erythrocyte sedimentation rate; CRP: C-reactive protein; and IgG: immunoglobulin G.
